# Writing a Case Report or Case Series: Practical Guidance for Clinicians and Researchers

**DOI:** 10.1002/ccr3.73298

**Published:** 2026-08-07

**Authors:** Thomas Aaron Ricks, Roberto Berebichez‐Fridman

**Affiliations:** ^1^ School of Nursing, Midwifery & Paramedicine Australian Catholic University Fitzroy Victoria Australia; ^2^ Nurse Workforce Unit St Vincent's Hospital Melbourne Victoria Australia; ^3^ Associate Editor, Clinical Case Reports, Published by John Wiley & Sons; ^4^ Orthopaedic Surgery Department American British Cowdray Medical Center Mexico City Mexico; ^5^ Editor‐In‐Chief, Clinical Case Reports, Published by John Wiley & Sons

## 1 The Value of Case Reports and Case Series

Case reports and case series are valuable because they turn clinical encounters into clinical learning. They are grounded in real patient care and can communicate observations, decisions, practices, risks, and outcomes that may not be visible in larger studies [1]. A case report usually focuses on one patient and provides a detailed account of the clinical presentation, assessment, diagnosis, management, outcome, and teaching point arising from that case. Similarly, a case series includes a small number of related patients and is useful when the value lies in identifying shared patterns in presentation, complications, responses, or outcomes across cases.

Many case reports and case series begin with something clinically noteworthy, such as an unusual presentation, an uncommon diagnosis, an adverse event, an unexpected response to treatment, or a clinical course that differs from what would usually be expected in practice. Although these observations are important to report, rarity or novelty alone is not sufficient justification for publication and does not, in itself, make a case report or case series valuable. Cases that illustrate common conditions, typical presentations, or everyday clinical challenges can be equally important when they highlight clinical reasoning, decision‐making, pitfalls in practice, or near‐misses that reveal how errors were avoided or could have occurred. Ultimately, the value of the case report or case series lies in the teaching point it offers to readers.

A useful case report or case series should help clinicians, including those from other specialties or health disciplines, recognize a clinical pattern, consider an alternative diagnosis or approach to management, understand clinical reasoning, or avoid diagnostic and management errors. Strong case reports and case series move beyond description. They offer readers a clear clinical lesson and prompt reflection on how that lesson may inform future decision‐making and practice [2].

## 2 Case Reports and Case Series Within the Hierarchy of Evidence

Evidence hierarchies help clinicians, researchers, and readers understand the relative strength of different forms of evidence. Within the Joanna Briggs Institute (JBI) levels of evidence, case reports and case series are positioned as Level 4 evidence within observational–descriptive studies [3]. This position within the evidence hierarchy should not diminish the value of case reports and case series; rather, it clarifies their purpose. These reports can describe clinically meaningful observations, highlight unusual or important presentations, identify novel adverse events or complications, suggest early clinical signals, and generate hypotheses for further research. In some instances, observations first described in case reports or case series may later be examined through higher‐level study designs, such as case–control studies, cohort studies, or clinical trials [1].

However, case reports and case series should not be written as though they establish causation, treatment effectiveness, or safety, prevalence, diagnostic accuracy, or generalisability. They are best understood as descriptive and hypothesis‐generating forms of evidence rather than definitive or practice‐changing evidence on their own.

## 3 From Clinical Observation to Teaching Point

A clear teaching point or a teaching message should be identified before writing a case report or case series. In *Clinical Case Reports*, this teaching point is expressed as a Key Clinical Message (KCM) [4]. While a clinical observation may prompt the report, the teaching point gives the manuscript its direction. It helps authors move from describing what happened in practice to explaining why the case or cases are meaningful for others. The teaching point should be specific, clinically meaningful, useful, and proportionate to the level of evidence. It should also be understandable to readers who may not be from the same clinical specialty area or health discipline. A teaching point may arise from different parts of the patient care journey, including the presentation, diagnostic process, differential diagnosis, treatment or management decisions, an innovative therapeutic approach, clinical outcomes, adverse events or complications, and patient‐centred, ethical, or interprofessional aspects of care.

Non‐specific or generic teaching points that are not clearly linked to the details of the reported case or cases, or do not add meaningful insight beyond general knowledge, are less useful. Statements such as “clinicians should be aware of this condition,” “this is a rare condition,” or “further research is needed” do not provide specific or practical guidance for readers. Stronger teaching points explain what clinicians should recognize, consider, question, or reflect on in similar clinical situations. The KCM should distil the teaching point into a clear and practical message that promotes reflection on clinical reasoning, decision‐making, patient care, or future practice, while remaining proportionate to what the case report or case series can support.

## 4 Reporting the Clinical Story With Clarity and Completeness

A well‐written case report or case series should present the clinical story in a way that readers can follow. The aim is not to include every possible detail but to provide a sufficiently complete clinical picture so readers can understand the patient context, clinical reasoning, management, outcome, and teaching point. It can be helpful to think of this as telling the story to another health professional who has never met or cared for the patient and who may be from a different specialty or discipline.

Established reporting frameworks, such as the CARE (CAse REport) guidelines [5], can help authors ensure that important elements are not missed. The CARE guidelines provide a general structure for improving the completeness and transparency of case report writing, while the modified CARE checklist used by Clinical Case Reports [4] (see Table 1) helps authors meet the journal's specific reporting and submission requirements. These frameworks should not make reports formulaic; rather, they help ensure that the clinical story includes the information readers need to understand what happened, why decisions were made, and what lessons can be drawn from the report.

**TABLE 1:** Modified CARE checklist.

Checklist item description	Present in the manuscript (Yes/No) – page number
Title: Clearly reflects the case	
2 Key Clinical Message: One clear take‐home message, ≤ 50 words	
3 Abstract (optional): Unstructured, no subheadings.	
4 Keywords: 4–6 relevant keywords	
5 Introduction: Briefly explain why the case is unique (include key references).	
6 Patient Information De‐identified patient details Main symptoms/concerns Medical, family, psychosocial, and genetic history (if relevant) Prior interventions and outcomes	
7 Clinical Findings: Key physical exam and clinical findings.	
8 Timeline: Chronological summary (table/figure encouraged).	
9 Diagnostic Assessment Tests performed (labs, imaging, etc.) Diagnostic challenges Final diagnosis with differentials Prognosis (if relevant)	
10 Therapeutic Intervention Type (medical, surgical, etc.) Details (dose, duration) Changes and rationale	
11 Follow‐up and Outcomes Clinical and patient‐reported outcomes Follow‐up results Adherence/tolerance Adverse events	
12 Discussion Strengths and limitations Comparison with literature Rationale for conclusions	
13 Conclusion: Final take‐home message (1 short paragraph, no references)	
14 Ethics & Consent Confirm informed consent was obtained Full patient anonymization along the manuscript	
15 Length Case reports: 1000–3000 words Case images: ≤ 500 words Case videos: ≤ 100 words	
16 References Case reports: no limit Case images: ≤ 3 references Case videos: ≤ 2 references No specific format but use a consistent format throughout the manuscript	
17 Case Series (if applicable): Maximum of 3 cases	
18 Letters to the Editor Written in response to a manuscript that has been published in Clinical Case Reports ≤ 3000 words	
19 Figures High‐quality images only No photos of imaging screens Ensure full patient anonymization Add relevant imaging studies, histopathological results, intraoperative pictures (when applicable), or other diagnostic studies	

The report should describe the clinical presentation, including the patient's main signs and symptoms, concerns, and reason for seeking care. It should also include relevant clinical background, such as medical, family, psychosocial, or genetic history and prior interventions or outcomes, where these help readers understand the case. The clinical assessment should be reported clearly, including observations, physical examination findings, and other relevant assessments. The diagnostic process should be described, including investigations, diagnostic challenges (if applicable), differential diagnoses, and the final diagnosis. Comprehensive clinical documentation may include relevant laboratory results, imaging, histopathology, procedural or intraoperative findings, and other diagnostic information needed to understand the report. Where imaging, histopathology, or procedural findings are central to the KCM, authors should consider including high‐quality original figures alongside their written interpretation. Where diagnostic or management decisions are based on established evidence, guidelines, or accepted clinical practice, authors should identify and cite relevant sources to show the evidence underpinning those decisions. Treatment or management should be described with sufficient detail, including the type of intervention, dose or intensity, timing, duration, any modifications made, and the rationale for those modifications, where relevant. Follow‐up and outcomes should be reported, including clinical outcomes, patient‐reported outcomes, tolerance, adherence, and adverse events where applicable. Follow‐up should be sufficient to evaluate clinically meaningful outcomes, late complications, and, where relevant, the durability of improvement. The appropriate duration will depend on the condition, intervention, and KCM. For example, in some orthopedic or surgical reports, approximately 12 months of follow‐up may be appropriate, whereas a shorter period may be justified in other clinical contexts. A chronological structure, such as a timeline, can help readers understand how the case evolved.

Factual clinical information should be reported clearly rather than replaced by vague interpretation. For example, rather than writing that “the patient's vital signs were unremarkable,” authors should provide the relevant vital signs. Similarly, rather than stating only that “the electrocardiogram (ECG) showed normal sinus rhythm,” authors should report the relevant ECG findings. This allows readers to understand the clinical context and interpret the significance of the findings for themselves.

## 5 Interpreting the Case in the Discussion

The discussion places the reported clinical observations within current knowledge and explains their significance for clinical practice. Rather than repeating the case description, it should interpret the principal findings and connect them to the KCM.

The discussion may begin with a concise synthesis of the patient's presentation, diagnosis, management, and outcome, without duplicating details already reported. It should then explain how the case or series contributes to current understanding. A central component of the discussion is a comparison with relevant published literature [6]. Authors should identify similarities and differences in clinical presentation, diagnostic assessment, management, and outcomes, drawing on relevant, current, and high‐quality literature rather than relying primarily on isolated or outdated sources. The discussion should also explain the clinical reasoning underpinning important diagnostic, and management decisions and whether these decisions aligned with or differed from available evidence, guidelines, or accepted practice. Where relevant, authors may consider the underlying mechanisms that could explain the presentation and clinical course. Evidence‐supported interpretations should be distinguished clearly from hypotheses or speculation.

The clinical implications should be linked directly to the KCM and explicitly discussed. Authors should explain how the case or cases reinforce, challenge, or extend current understanding and identify lessons relevant to similar clinical situations. Case reports and case series may inform clinical reasoning and practice. However, any recommendations should remain proportionate to the reported evidence and should avoid drawing conclusions that extend beyond the reported data.

Specific limitations that affect interpretation should also be acknowledged, such as missing clinical information, incomplete follow‐up, unavailable investigations, or uncertainty about causal attribution. The inclusion of a single patient in a case report, or a small number of related patients in a case series, is an inherent feature of the design rather than, by itself, a limitation. Acknowledging relevant limitations enhances the credibility of the report and helps readers interpret the findings appropriately.

## 6 Ethical Considerations

Case reports and case series involve one patient or a small number of patients, making the protection of privacy and confidentiality particularly important [7]. Even after direct identifiers, such as the patient's name, initials, hospital identification number, or date of birth, have been removed, the patient may still be re‐identified from the combination of clinical and contextual details reported. Authors should therefore assess the risk of re‐identification across the manuscript as a whole. Information such as age, occupation, geographical location, family circumstances, diagnosis, unusual presentation, clinical course, or treatment may enable recognition by relatives, colleagues, members of the local community, or healthcare professionals involved in the patient's care. This risk may be particularly high for rare or distinctive cases [8]. Clinically important information should be retained, but unnecessary precision or personal detail should be omitted, generalized, or presented differently where this reduces the risk of re‐identification without compromising the meaning of the report.

Written informed consent for publication should be obtained for each patient included in a case report or case series before submission. Consent for clinical care or participation in research does not necessarily constitute consent for publication. Patients should understand what clinical information, images, or other media will be published, that the report may be openly accessible worldwide, and that complete anonymity cannot always be guaranteed despite efforts to de‐identify the information. Where the patient cannot provide consent, such as a child, an adult lacking decision‐making capacity, or a deceased patient, consent should be managed in accordance with applicable legal, institutional, and journal requirements and may need to be provided by a parent, legal guardian, next of kin, or another authorized representative. Additional safeguards may be required where vulnerability or power imbalances could affect whether consent is freely given. Obtaining consent does not remove the authors' continuing responsibility to protect the patient's privacy and confidentiality [8].

Images, audio recordings, videos, and other media may contain identifying information even when names or other written identifiers have been removed. Authors should remove embedded identifiers and assess whether faces, voices, tattoos, birthmarks, or other distinctive features could reveal the patient's identity. Masking only the eye region is not sufficient to ensure anonymity. Any editing used to protect identity should preserve the accuracy and integrity of the underlying clinical information. Genetic information, family pedigrees, and inherited conditions may also identify or affect relatives, and additional consent may therefore be required.

Formal ethics approval is not universally required for case reports or small case series; however, requirements vary across institutions, jurisdictions, and clinical contexts. Authors should check whether ethics approval is required by their institution or jurisdiction. Advice from an ethics committee or institutional review board should be sought when requirements are unclear, when patients may be vulnerable, or when the work extends beyond routine clinical care and case reporting. Any ethics approval, exemption, or waiver obtained should be stated clearly in the manuscript. Ethics approval and informed consent for publication are separate requirements, and neither substitutes for the other. Ethical responsibilities extend beyond consent and confidentiality. Case reporting should respect patient autonomy and dignity, minimize the risk of physical, psychological, social, or reputational harm, avoid coercion or exploitation, and represent patients accurately and respectfully using bias‐free and culturally appropriate language [7, 8].

## 7 Common Features of Case Reports and Case Series May Be Rejected

Case reports and case series may be unsuitable for publication when their contribution to clinical knowledge or learning is not sufficiently clear. Authors may perceive a clinical encounter as unique because it is the first time they have encountered it in their own practice; however, a review of the published literature may show that similar reports already exist. Before preparing a manuscript, authors should therefore determine whether the report offers a distinct KCM or a meaningful perspective beyond what is already known. Although the condition, diagnosis, presentation, or treatment does not need to be entirely new, the manuscript should provide new clinical insight, challenge or refine an existing concept, illustrate a diagnostic or therapeutic dilemma, or reinforce an important but underrecognized lesson. This may also be achieved through a common condition or routine clinical situation when the report contributes meaningfully to clinical reasoning, patient care, or reflective practice.

Other common reasons a manuscript may be unsuitable for publication include poor alignment between the clinical account, discussion, conclusion, and KCM; incomplete or inconsistent clinical documentation; an unclear chronology; insufficient follow‐up for the outcomes being reported; poor‐quality or uninformative figures; overinterpretation of the findings; and inadequate comparison with current literature. Failure to meet ethical, reporting, or journal requirements may also prevent a manuscript from progressing, regardless of the potential value of the underlying clinical encounter. Authors should therefore ensure that the report is coherent, adequately supported, and consistent with the relevant reporting and submission requirements.

## 8 Conclusion

Case reports and case series transform individual clinical experiences into shared knowledge. Their contribution depends not on rarity alone, but on a clear KCM, complete and transparent clinical reporting, balanced interpretation within the context of current literature, and ethical respect for the patients whose experiences are described. When these principles are followed, case reports and case series can strengthen clinical reasoning, support reflective practice, identify important clinical observations, and generate questions for future research. In doing so, they can meet publication standards while making meaningful contributions to clinical knowledge and patient care.

## Author Contributions

**Thomas Aaron Ricks:** conceptualization, methodology, writing – original draft, writing – review and editing, project administration, resources. **Roberto Berebichez‐Fridman:** supervision, conceptualization, writing – original draft, writing – review and editing, methodology, resources.

## Funding

The authors have nothing to report.

## Conflicts of Interest

Thomas Aaron Ricks declares serving as an Associate Editor for Clinical Case Reports; Roberto Berebichez‐Fridman declares serving as the Editor‐in‐Chief for Clinical Case Reports.

## Data Availability

Data sharing not applicable to this article as no datasets were generated or analysed during the current study.
